# Maternal epigenetic clocks measured during pregnancy do not predict gestational age at delivery or offspring birth outcomes: a replication study in metropolitan Cebu, Philippines

**DOI:** 10.1186/s13148-022-01296-6

**Published:** 2022-06-22

**Authors:** Calen P. Ryan, Raviraj J. Rege, Nanette R. Lee, Delia B. Carba, Michael S. Kobor, Julie L. MacIsaac, David S. Lin, Parmida Atashzay, Christopher W. Kuzawa

**Affiliations:** 1 Department of Epidemiology, Robert N. Butler Columbia Aging Center, Columbia University Mailman School of Public Health, Columbia University, New York, NY 10032 USA; 2 Department of Anthropology, Northwestern University, Evanston, IL 60208 USA; 3 USC-Office of Population Studies Foundation, University of San Carlos, Talamban, Cebu City, Philippines; 4 Department of Medical Genetics, Faculty of Medicine, University of British Columbia, Vancouver, Canada; 5 BC Children’s Hospital Research Institute, University of British Columbia, Vancouver, Canada; 6 Centre for Molecular Medicine and Therapeutics, Vancouver, Vancouver, BC Canada; 7 Institute for Policy Research, Northwestern University, Evanston, IL 60208 USA

**Keywords:** DOHaD, Aging, Pregnancy, Epigenetic clocks, Senescence

## Abstract

Adverse birth outcomes, such as early gestational age and low birth weight, can have lasting effects on morbidity and mortality, with impacts that persist into adulthood. Identifying the maternal factors that contribute to adverse birth outcomes in the next generation is thus a priority. Epigenetic clocks, which have emerged as powerful tools for quantifying biological aging and various dimensions of physiological dysregulation, hold promise for clarifying relationships between maternal biology and infant health, including the maternal factors or states that predict birth outcomes. Nevertheless, studies exploring the relationship between maternal epigenetic age and birth outcomes remain few. Here, we attempt to replicate a series of analyses previously reported in a US-based sample, using a larger similarly aged sample (*n* = 296) of participants of a long-running study in the Philippines. New pregnancies were identified prospectively, dried blood spot samples were collected during the third trimester, and information was obtained on gestational age at delivery and offspring weight after birth. Genome-wide DNA methylation was assessed with the Infinium EPIC array. Using a suite of 15 epigenetic clocks, we only found one significant relationship: advanced age on the epigenetic clock trained on leptin predicted a significantly earlier gestational age at delivery (*β* = − 0.15, *p* = 0.009). Of the other 29 relationships tested predicting gestational age and offspring birth weight, none were statistically significant. In this sample of Filipino women, epigenetic clocks capturing multiple dimensions of biology and health do not predict birth outcomes in offspring.

**Supplementary Information:**

The online version contains supplementary material available at 10.1186/s13148-022-01296-6.

## Introduction

Birth outcomes like birth weight, length, and gestational age predict both short- and long-term health. For example, early gestational age at birth predicts the two largest causes of death in premature infants: underdevelopment of mature organs and bronchopulmonary dysplasia, a chronic lung disease that damages alveolar tissue [1, 2]. In addition, the field of the Developmental Origins of Health and Disease (DOHaD) has established that being born early and small for gestational age also predicts elevated long-term risk for developing respiratory conditions like idiopathic lung disease and chronic metabolic diseases like hypertension, diabetes, and other cardiovascular diseases [2–6]. Experimental work with animal models shows that restricting prenatal nutrition, or imposing acute stress during pregnancy, replicates many of these long-term outcomes in offspring, showing that gestational conditions can have lasting effects on health in the next generation [7, 8].

While nutrition has received broadest attention for its role in fetal growth, there is growing evidence that the mother’s physiology and metabolism, including systems like stress physiology and inflammation, can impact fetal growth and development operating through effects on gestational conditions like nutrient delivery, oxidative stress, or exposure to metabolic or other hormones [9]. As a result, disturbances in the normal levels and amounts of exposure of these biological effectors can result in altered function and long-term disease risk [10]. As a common example, dysregulation of the hypothalamic–pituitary (HPA) axis during pregnancy is associated with increased levels of maternal cortisol, which elevates risks for premature delivery and low birth weight and can cross the placenta to have direct “programming” effects on fetal metabolism and physiology [11, 12]. Hypertension has been shown to lead to lower birth weights, likely operating through factors like altered blood flow, along with the common co-occurrence of elevated inflammatory cytokines that can suppress growth [13, 14]. Conversely, dysregulated glucose homeostasis, as reflected in uncontrolled diabetes during pregnancy, increases delivery of glucose across the placenta, and can lead to larger than expected newborns with elevated risk of developing obesity and diabetes in as adults [15, 16].

A set of tools called epigenetic clocks have recently been shown to reflect various domains of physiology and metabolism, and thus could be useful for gauging the intergenerational impacts of chronic maternal physiological and metabolic dysregulation [17, 18]. Epigenetic clocks are calculated using predictable age-related changes in the epigenome—particularly methylation of cytosine-phosphate-guanine (CpG) sites on DNA or DNA methylation (DNAm). Although commonly used epigenetic clocks are notable for their ability to predict one’s chronological age [19, 20], individuals who appear epigenetically older than their chronological age, known as epigenetic age acceleration, tend to have increased risk for future morbidity and shorter life expectancies. Other clocks have been trained to predict suites of clinical markers and are particularly powerful predictors of life expectancy and the pace of biological aging [21–23].

Since epigenetic clocks can be trained on effectively any set of metabolic/physiological processes or states, they are powerful tools for characterizing these states [18]. For the purposes of clarifying the intergenerational determinants of birth outcomes, they provide integrative, summary information on a mother’s metabolic and physiological state and thus allow an assessment of the impact of these maternal experiences on the next generation. Despite this promise, to date few studies have investigated the relevance of epigenetic clocks, capturing different domains of maternal biology and health, as predictors of offspring birth outcomes. One study, conducted among women in California (*n* = 77), evaluated 15 epigenetic clocks as predictors of gestational age at birth and birth weight, and found mixed evidence that advanced maternal epigenetic age predicted early gestational age at birth and low birthweight in offspring, suggesting to the authors that epigenetic age may be predictive of adverse fetal outcomes [24].

In the present paper, we seek to replicate this analysis by examining relationships between the same suite of 15 epigenetic clocks, measured in DNA obtained from blood during pregnancy, and prospectively measured birth outcomes in the offspring of those pregnancies. Data come from the Cebu Longitudinal Health and Nutrition Survey (CLHNS), a cohort study that has followed a large, diverse sample of women and their offspring in metropolitan Cebu City, Philippines for nearly four decades [25]. The present analyses focus on pregnancies of 296 expecting female young adults and their newborns born between 2009 and 2014. The 15 published epigenetic clocks that we focus on provide complementary information on multiple dimensions of the mother’s chronic biological dysregulation, and replicate those previously investigated [24]. Clocks included two first-generation epigenetic clocks trained on chronological age [19, 20], two second-generation clocks trained on clinical biomarkers and mortality risk [22, 23] and 11 clocks trained on clinical biomarkers that are themselves linked with morbidity and mortality [23, 26]. We hypothesized that advanced maternal epigenetic age acceleration based upon these indices would predict adverse fetal outcomes, as reflected in decreased gestational age and measured weight at birth.

## Methods

### Study sample and design

Data come from the Cebu Longitudinal Health and Nutrition Survey (CLHNS), a longitudinal survey of 3,080 infants and their mothers who were recruited during their pregnancies between 1983 and 1984 in Metropolitan Cebu, Philippines [25]. Out of the 1447 original female cohort infants, 823 were interviewed in a later 2009 survey (at ages 25–26). This additional survey tracked new pregnancies among these women between 2009 and 14. There were 383 who reported pregnancies (28% with 2–3 pregnancies) within the tracking period, yielding 507 pregnancies. Women were visited in-home during pregnancy for anthropometric and questionnaire assessments, along with collection of a dried blood spot (DBS)—capillary whole blood collected on filter paper—for DNAm measurement. A second visit was arranged soon after delivery to obtain additional information from the mothers and to measure anthropometry in their newborns. Body weight was measured in-home by trained interviewers using standardized procedures [27] as soon after birth as possible, with a mean age of 4 days after data cleaning (described below). All research was conducted under conditions of written informed consent, and with approval of the Institutional Review Boards of Northwestern University (Evanston, Illinois), and the Office of Population Studies Foundation (Cebu, Philippines).

### Variable construction

A composite score of socioeconomic status was measured as a combination of income, education, and assets. Participants reported their annual income from all sources, including in-kind services, and the sale of livestock or other products by household members during the prior year, which were summed to determine total household income. Incomes were log-transformed. Maternal education (in years) was also reported. Participants also reported on ten assets (coded 0, 1) that were selected to capture population-relevant aspects of social class, including electricity, refrigerators, air conditioners, color televisions, cable TV, tape recorders, electric fans, jeepneys, cars, trucks, and owning their residence. In addition, house construction type (i.e., light, mixed, permanent structure) was coded as 1, 2, or 3, respectively. Thus, asset scores ranged from 0 to 13. A principal components analysis was run on log income and assets, along with maternal education, at sample collection. The first dimension explained 70% of the variation in our composite SES-score, and individual scores for the top component of variation were used as our measure of SES.

Because women were enrolled in the birth outcome sub-study after they were pregnant, we used height and weight measurements collected during prior surveys to estimate pre-pregnancy BMI. We used 2009 BMI when available, and then used 2007 and 2005 data as necessary. Under the assumption that women will tend to maintain a stable position within the population BMI distribution even as the population mean increases with age, we converted all BMIs to age-specific within-sample Z-scores before pooling into a single pre-pregnancy BMI variable. Supporting the validity of this approach, the correlation between Z-scores for BMI measured in 2005 and 2009 was very high (*r* = 0.84). Offspring gestation age was calculated using the time between the last reported menstrual period and infant birth date. Days pregnant at maternal blood sampling was determined by subtracting the time between the blood sample and infant birth date from gestation age. Descriptive statistics of anthropometric measurements and other covariates are provided in Table 1.

**Table 1:** Descriptive statistics for mothers in the study

Characteristic	*N* = 296^1^
Maternal age at measurement	27.82 (24.99, 30.79)
Days pregnant at measurement	207 (160, 288)
Current smoker?	17 (5.7%)
Grade completed	11.2 (2.0, 22.0)
SES z-score	0.06 (− 3.32, 5.10)
Pre-pregnancy BMI z-score	0.02 (− 1.89, 3.90)
*Pregnancy number*	
1	41 (14%)
2	87 (29%)
3	67 (23%)
4	52 (18%)
5	25 (8.4%)
6 +	23 (8.1%)

^1^Mean (range); *n* (%)

### Sample inclusion criteria

DNAm was measured in a total of 334 women and only women with complete information for all variables were included. For each woman, the last pregnancy during the 2009–2014 tracking period was used unless inadequate DBS sample remained, in which case a blood sample from the prior pregnancy was used. Fifteen women were missing pre-pregnancy BMI, 2 women were missing data on offspring developmental outcomes, and DNAm for one woman did not pass quality control; these women were therefore excluded. Analyses were further limited to women with newborns with gestational ages between 32 and 44 weeks, which excluded 5 very premature births, 10 individuals with implausibly late deliveries, and 2 women for whom gestational age data were missing. To minimize impacts of the infant’s environment and growth after birth, analyses of infants were also limited to those measured within 2 weeks of birth, with postnatal weight models adjusting for age at measurement (4 individuals measured more than 2 weeks after birth were excluded). After all exclusions, the final sample with all necessary biological and questionnaire data included 296 women singleton births with complete information.

### DNA methylation sample processing and epigenetic clock calculation

DNA was extracted from 1 to 3 dried blood spots (DBS) using a standard protocol; purified DNA was concentrated using a Vacufuge Plus vacuum concentrator (Eppendorf), then treated with sodium bisulfite (Zymo EZDNA, Zymo Research, Irvine, CA, USA), and quantified by nanodrop, and 160 ng of converted DNA was applied to the Illumina Infinium MethylationEPIC BeadChip under standard conditions (Illumina Inc., San Diego, CA). Technicians were blind to any information regarding participant characteristics, and samples were randomly assigned to plate, chip, and row. Background subtraction and color correction were performed using Illumina Genome Studio with default parameters. Data were then exported into R for further analysis. Quality control involved first confirming participant sex and replicate status. This was followed by quantile normalization using lumi on all probes including SNP-associated and XY multiple binding probes. To maximize the number of sites available for the epigenetic age calculator, probes with detection p values above 0.01 were called NA for poor performing samples only and were otherwise retained [18].

Epigenetic age for all clocks was calculated using the online calculator (http://labs.genetics.ucla.edu/horvath/dnamage/). Background-corrected beta values were processed further using the calculator’s internal normalization algorithms. Clocks included were: Horvath’s epigenetic age (DNAmAge) [20], intrinsic epigenetic age acceleration or DNAmIEAA [20, 28], extrinsic epigenetic age acceleration or DNAmEEAA [19, 28], DNAmPhenoAge [22], DNAmTL [26], senescent T-cell age, DNAmGrimAge [23], and the clocks that make up the DNAmGrimAge clock (DNAm PAI-1, DNAm ADM, DNAm, B2M, DNAm cystatin C, DNAm GDF, DNAm leptin, DNAm TIMP1, and DNAm smoking pack years)[23]. DNAmIEAA examines the intrinsic biological age of immune cells but does not depend on age-related changes in immune cells in the blood [28]. DNAmEEAA captures immune cell biological age due to both intrinsic immune cell age and changes in immune cell populations in the blood [28]. DNAmPhenoAge is determined using the Levine Method, which uses sites selected due to associations with phenotypic age indicators and chronological age [22]. DNAmGrimAge is a marker enriched for DNA methylation sites that are surrogate markers for blood plasma proteins related to mortality. These include DNAmPAI1 (Plasminogen Activator Inhibitor-1), DNAmADM (Adrenomedullin), DNAmB2M (Beta-2-Microglobulin), DNAmCystatinC (Cystatin C), DNAm GDF (Growth Differentiation Factor-15), DNAm leptin (Leptin), DNAm TIMP1 (TIMP Metallopeptidase Inhibitor-1), and DNAm smoking pack years serve as these surrogate DNA methylation markers [23]. DNAmTL is a surrogate methylation measure of leukocyte telomere length. In all cases, maternal epigenetic age acceleration, the residual of epigenetic age on chronological age (as well as days since conception at the time of blood sample and smoking status), was used as predictor of interest.

### Statistical analysis

We first ran descriptive statistics before running a sequence of multiple linear regression models designed to assess relationships between maternal epigenetic age acceleration and two fetal outcomes (gestational age and measured weight after birth). Models predicting gestational age adjusted for offspring sex, a composite score of socioeconomic status, and pre-pregnancy body mass index (BMI) z-scores. Postnatal outcomes were adjusted for days after birth of anthropometry measurement and gestational age at birth and our composite socioeconomic status score. Since we are replicating prior work that did not correct for multiple testing, we did not correct for multiple comparisons in our main analysis. For transparency, however, we also point out results that differed using a Bonferroni adjusted alpha of 0.05/15 = 0.003. Furthermore, we ran sensitivity analyses that included an additional term for chronological age (beyond that residualized in the epigenetic age acceleration measures), pregnancy order, cell counts (CD4T, CD8T, monocyte, granulocyte, NK cell, B cell), and batch effects (batch, plate, chip, row). All statistical analyses were conducted using R version 4.0.4.

## Results

The women in our study ranged between 25 and 30.8 years at the time of the study (mean age = 27.8 years old). Blood spots for DNAm were taken between 160 and 288 days into pregnancy, with a mean gestational timing of 207 days. Education ranged from 2 to 22 years (22 equivalent to an MD, law degree, or priesthood), and 17 women smoked. Over 16% of the women in the study had experienced 5 or more pregnancies, while 57% had experienced at least 3 pregnancies. Descriptive statistics of these and other maternal covariates are provided in Table 1.

Slightly more infants were categorized as male (52%), with a mean gestational age at birth of 277 days. Postnatal measurement occurred between 1 and 14 days after birth, with the mean age at measurement of roughly 4 days. Descriptive statistics of infant weight, length, and other anthropometric measures are provided in Table 2.

**Table 2:** Descriptive statistics for infant outcomes

Characteristic	*N* = 296^1^
Infant sex	
Female	141 (48%)
Male	155 (52%)
Gestational age (days)	39.6 (32.4, 44)
Postnatal measurement age (days)	4.0 (1, 14)
Weight (kg)	3.08 (1.68, 4.30)

^1^n (%); Mean (range)

**Fig. 1:**
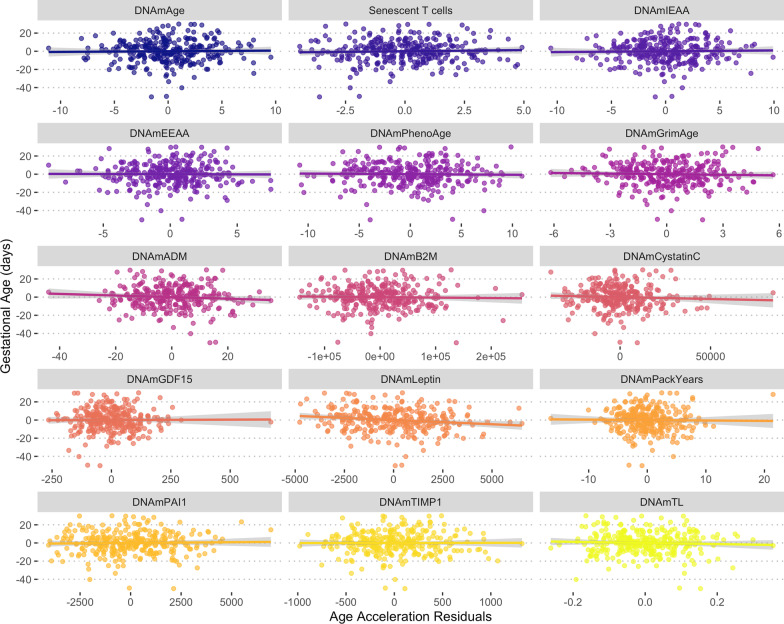
Relationships between maternal epigenetic age acceleration during pregnancy for 15 epigenetic clocks and offspring gestational age. Epigenetic clock residuals after controlling for maternal chronological age, days post-conception at the time of blood sampling, and smoking status. Additional model summary output provided in Table 3 and Additional file 2: Table S1

**Fig. 2:**
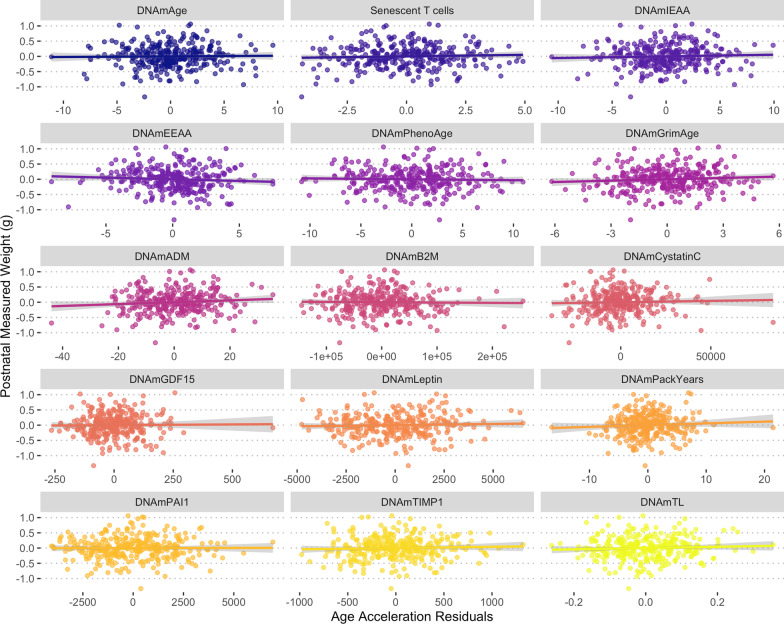
Relationships between maternal epigenetic age acceleration during pregnancy for 15 epigenetic clocks and offspring postnatal weight. Epigenetic clock residuals after controlling for maternal chronological age, days post-conception at the time of blood sampling, and smoking status. Additional model summary output provided in Table 3 and Additional file 2: Table S2

We found very little evidence that any of the 15 maternal clocks we examined were associated with either gestational age or postnatal weight (Table 3, Figs. 1 and 2, Additional file 2: Tables S1–S2). Of the relationships investigated, only the DNAmLeptin clock was significantly and negatively associated with gestational age (*p* = 0.009). This effect was not statistically significant using the Bonferroni adjusted threshold of 0.003. Our results were unchanged in sensitivity analyses, which included an additional term for age (in addition to that in the epigenetic age acceleration measure) and pregnancy order (Additional file 2: Table S3–S4), cell counts (CD4T, CD8T, monocyte, granulocyte, NK cell, B cell) (Additional file 2: Table S5–S6), and batch effects (batch, plate, chip, row) (Additional file 2: Table S7–S8). A comparison between previously reported effects and our findings is provided in Additional file 1: Fig. S1.

**Table 3:** Summary results for regression models predicting gestational age at delivery and offspring birth weight using epigenetic age acceleration^a^

Outcome	Predictor	Std. *β*	Std. 95% CI	Test statistic	*P* value
*Gestational age*	DNAmAge	0.02	− 0.10–0.13	0.32	0.748
	Senescent T-cells	0.04	− 0.08 – 0.15	0.66	0.51
	DNAmIEAA	0.02	− 0.09–0.14	0.4	0.687
	DNAmEEAA	− 0.01	− 0.12–0.11	− 0.12	0.901
	DNAmPhenoAge	− 0.02	− 0.14–0.09	− 0.35	0.726
	DNAmGrimAge	− 0.04	− 0.15–0.08	− 0.62	0.539
	DNAmADM	− 0.08	− 0.20–0.03	− 1.4	0.163
	DNAmB2M	− 0.03	− 0.14–0.09	− 0.44	0.657
	DNAmCystatinC	− 0.05	− 0.17–0.06	− 0.88	0.378
	DNAmGDF15	0.01	− 0.11–0.12	0.13	0.899
	DNAmLeptin	− 0.15	− 0.26–− 0.04	− 2.63	0.009
	DNAmPackYears	− 0.02	− 0.13–0.10	− 0.26	0.797
	DNAmPAI1	0.03	− 0.09–0.15	0.46	0.643
	DNAmTIMP1	0.00	− 0.11–0.12	0.06	0.951
	DNAmTL	− 0.05	− 0.16–0.07	− 0.82	0.411
*Postnatal weight*	DNAmAge	0.02	− 0.09–0.12	0.3	0.765
	Senescent T-cells	0.05	− 0.06–0.16	0.89	0.375
	DNAmIEAA	0.04	− 0.06–0.15	0.82	0.415
	DNAmEEAA	− 0.07	− 0.18–0.04	− 1.31	0.192
	DNAmPhenoAge	− 0.03	− 0.14–0.08	− 0.55	0.582
	DNAmGrimAge	0.08	− 0.03–0.19	1.42	0.155
	DNAmADM	0.09	− 0.02–0.20	1.64	0.102
	DNAmB2M	− 0.02	− 0.12–0.09	− 0.31	0.757
	DNAmCystatinC	0.03	− 0.07–0.14	0.63	0.532
	DNAmGDF15	0.01	− 0.10–0.12	0.23	0.819
	DNAmLeptin	0.04	− 0.07–0.15	0.74	0.461
	DNAmPackYears	0.06	− 0.05–0.17	1.09	0.278
	DNAmPAI1	0.01	− 0.11–0.12	0.09	0.929
	DNAmTIMP1	0.04	− 0.07–0.15	0.71	0.479
	DNAmTL	0.05	− 0.06–0.16	0.96	0.339

^a^All models adjust for maternal age (are age acceleration measures), offspring sex, composite socioeconomic score, and the mother’s pre-pregnancy BMI; models predicting birth weight also adjust for gestational age at delivery and postnatal age of anthropometry measurement

## Discussion

In this study of women in metropolitan Cebu, Philippines, a panel of 15 epigenetic clocks chosen to replicate an analysis recently published in this journal, using a sample roughly 4 times larger, generally failed to predict birth outcomes or gestational age at delivery. Only a single clock—DNAmLeptin—predicted gestational age at delivery, with the other 29 relationships investigated not significant. These findings suggest that epigenetic clocks measured in pregnant mothers are not strong predictors of offspring birth outcomes, or could point to population variation in these relationships.

Of the 30 relationships that we evaluated, only DNAmLeptin predicted gestational age at delivery, a finding that has not been previously reported but which is directionally consistent with other work [24]. Leptin is a peptide hormone secreted from white adipocytes but also fetal and placental tissues and is an important regulator of food intake and energy expenditure [29] During pregnancy, leptin is involved in placentation and in regulation of maternal metabolic homeostasis [30]. Late pregnancy is associated with leptin resistance and elevated leptin levels, which are necessary to help meet the energetic requirements of the rapidly growing late-stage fetus. To the extent that DNAm leptin is a proxy of circulating leptin levels during pregnancy [23], the negative relationship between DNAm leptin and gestational age could be a compensatory increase in fetal leptin secretion in response to insufficient nutrient availability. Although we controlled for pre-pregnancy body mass index, higher leptin predicting gestational age might be expected to be particularly common in cases of maternal obesity, where pre-pregnancy leptin resistance can elevate baseline leptin levels and exacerbate pregnancy-induced leptin resistance.

To our knowledge, ours is the largest study linking commonly used epigenetic clocks with offspring birth outcomes to date, and the only one outside of affluent, Western settings where fertility tends to be low and outcomes like low birth weight relatively uncommon. For example, contrasting with prior work where primiparous women made up 61% of the sample [24], only 14% of women in our study were primiparous. Furthermore, our sample exhibited a great deal of variability in fertility, with more than half of the women in our study having been pregnant 3 or more times, and over 15% having had 5 or more pregnancies. Variation in fertility and study context are important because placentation and corresponding birth outcomes are affected by reproductive history [31, 32], and because epigenetic age varies across socioecological contexts [33]. These findings imply that while measures of maternal physiology, metabolism, and stress do seem to be associated with offspring epigenetic age acceleration [34, 35], maternal epigenetic age acceleration itself is not a good predictor of fetal and infant developmental outcomes.

Our study is not without limitations. We were not able to acquire reliable measures of birth weight immediately after birth due to the diversity of the sample, birth contexts, and geographic spread across the Cebu Metropolitan area. Thus, our measures of weight taken in infants are only proxies for outcomes measured at the time of birth. We minimized the potential for this to affect our results by including on infants measured within 2 weeks of birth, with a mean age of measurement of 4 days. This approach has the added benefit of all measurements being taken in triplicate by experienced staff using the same instruments and protocols. Another limitation was that our blood samples were not taken at the same time during pregnancy for each woman. This may be important because prior work has demonstrated that DNAm in general and epigenetic age specifically, and their relationship with birth outcomes, can change during pregnancy [36–38]. Nevertheless, our blood sampling occurred within a relatively narrow range of 23–41 weeks, and we adjusted all clock measures for gestational age at measurement. Finally, while our sample size of 296 is the largest yet to examine the relationship between maternal epigenetic clock age and fetal outcomes, a lack of statistical power could limit our ability to detect important biological relationships. Nevertheless, our power to detect previously reported effect sizes [24] given our sample size was greater than 0.99, suggesting that we were more than adequately powered to replicate previous findings.

In sum, our findings suggest that pregnancy measurement of epigenetic clocks that capture a range of biological pathways of pathophysiologic dysregulation and aging are not robust predictors of gestational age at delivery or offspring birth size. These findings fail to replicate recent work using an identical panel of clocks, and either point to a lack of consistent findings or population variation in these relationships.

## Supplementary Information

**Additional file 1: Fig. S1.** Comparison of findings from the current study to those of Ross et al. 2020. Colored circles and bars show the standardized beta estimates and standard errors from Ryan et al., whereas x’s indicate the standardized beta estimates for clocks that were significantly (red) or not significantly (gray) associated with birth outcomes in Ross et al. (errors not available for Ross et al.).

**Additional file 2.** Full model estimates for the relationship between maternal epigenetic age and gestation age and offspring post-natal weight. Estimates for models including birth order, cell count estimates, and batch, chip, and row are also provided.

## Data Availability

Longitudinal data are available for download at: https://dataverse.unc.edu/dataverse/cebu. Other data will be made available from the corresponding author upon reasonable request.
