# Supplementary material for: Maternal epigenetic clocks measured during pregnancy do not predict gestational age at delivery or offspring birth outcomes: a replication study in metropolitan Cebu, Philippines
Source: Clin Epigenetics. 2022 Jun 22;14:78. doi: 10.1186/s13148-022-01296-6 (PMC9219190; doi:10.1186/s13148-022-01296-6)
Supplement: Supplementary file 1 — Additional file 1: Fig. S1. Comparison of findings from the current study to those of Ross et al. 2020. Colored circles and bars show the standardized beta estimates and standard errors from Ryan et al., whereas x’s indicate the standardized beta estimates for clocks that were significantly (red) or not significantly (gray) associated with birth outcomes in Ross et al. (errors not available for Ross et al.). [file 13148_2022_1296_MOESM1_ESM.docx]

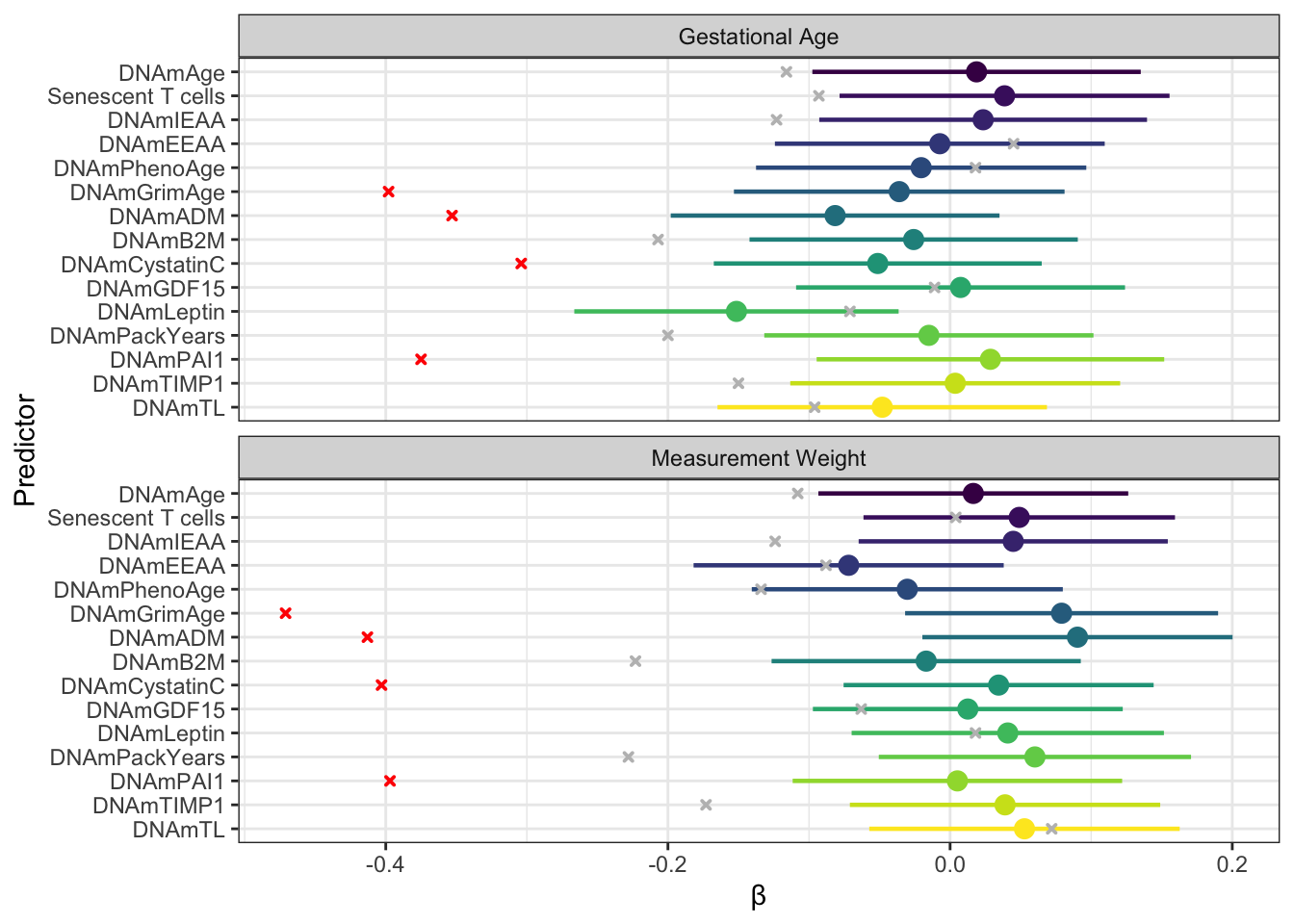


**Figure S1.** Comparison of findings from the current study to those of Ross et al. 2020. Colored circles and bars show the standardized beta estimates and standard errors from Ryan et al., whereas x’s indicate the standardized beta estimates for clocks that were significantly (red) or not significantly (gray) associated with birth outcomes in Ross et al. (errors not available for Ross et al.).
